# Tetramethylbenzidine: An Acoustogenic Photoacoustic Probe for Reactive Oxygen Species Detection

**DOI:** 10.3390/s20205952

**Published:** 2020-10-21

**Authors:** Roger Bresolí-Obach, Marcello Frattini, Stefania Abbruzzetti, Cristiano Viappiani, Montserrat Agut, Santi Nonell

**Affiliations:** 1 Institut Químic de Sarrià, Universitat Ramon Llull, Via Augusta 390, 08017 Barcelona, Spain; roger.bresoliobach@kuleuven.be (R.B.-O.); marcello.frattini7@gmail.com (M.F.); montserrat.agut@iqs.url.edu (M.A.); 2 Department of Chemistry, Katholieke Universiteit Leuven, celestijnenlaan 200F, 3001 Heverlee (Leuven), Belgium; 3 Dipartimento di Scienze Matematiche, Fisiche e Informatiche, Università di Parma, Parco Area delle Scienze 7A, 43124 Parma, Italy; stefania.abbruzzetti@unipr.it (S.A.); cristiano.viappiani@unipr.it (C.V.)

**Keywords:** 3,3′,5,5′-tetramethylbenzidine, reactive oxygen species (ROS), reactive nitrogen species (RNS), photoacoustic probes, optical sensors, singlet oxygen

## Abstract

Photoacoustic imaging is attracting a great deal of interest owing to its distinct advantages over other imaging techniques such as fluorescence or magnetic resonance image. The availability of photoacoustic probes for reactive oxygen and nitrogen species (ROS/RNS) could shed light on a plethora of biological processes mediated by these key intermediates. Tetramethylbenzidine (TMB) is a non-toxic and non-mutagenic colorless dye that develops a distinctive blue color upon oxidation. In this work, we have investigated the potential of TMB as an acoustogenic photoacoustic probe for ROS/RNS. Our results indicate that TMB reacts with hypochlorite, hydrogen peroxide, singlet oxygen, and nitrogen dioxide to produce the blue oxidation product, while ROS, such as the superoxide radical anion, sodium peroxide, hydroxyl radical, or peroxynitrite, yield a colorless oxidation product. TMB does not penetrate the *Escherichia coli* cytoplasm but is capable of detecting singlet oxygen generated in its outer membrane.

## 1. Introduction

Reactive oxygen species (ROS) are a family of oxygen-based molecules including singlet oxygen (^1^O_2_), superoxide radical anion (O_2_^•−^), hydrogen peroxide (H_2_O_2_), hydroxyl radical (^•^OH), and peroxyl radical (ROO^•^), as well as hypochlorous acid and hypochlorite anion (HClO and ClO^−^, respectively) [1]. In low concentrations, ROS are adventitiously produced in several metabolic processes of aerobic organisms [2], derived from molecular ground oxygen (^3^O_2_), and show high reactivity mainly as oxidant species. ROS oxidant effects are usually controlled by the cell’s antioxidant defense mechanism [3]. However, when such processes are insufficient or overwhelmed, an accumulation of free ROS takes place inside cells, which are readily reactive towards proteins, nucleic acids, and/or lipids [4]. Elevated ROS concentrations can lead to irreversible damage and eventually to cell death. In addition to ROS, reactive nitrogen species (RNS) present also a high oxidative reactivity towards biomolecules. RNS are a family of molecules derived from nitric oxide (^•^NO), such as nitrogen dioxide (^•^NO_2_), nitrite (NO_2_^−^), or peroxynitrite (ONOO^−^) [5]. RNS can act alone or together with ROS to damage cells, mainly exerting nitrosative stress [6]. ROS and RNS can also be generated by light, whereby a photoexcited molecule can initiate a cascade of energy- or electron-transfer processes leading to the formation of ROS/RNS [7]. Medical technologies, such as photodynamic therapy, take advantage of this effect to locally eliminate cancerous cells or pathogenic microorganisms [8,9]. 

Due to their important role in biological and medical processes, ROS/RNS detection techniques are required to monitor and quantify them with spatio-temporal resolution [10]. Currently available techniques can be broadly classified into two groups, one in which the analyte is directly monitored, e.g., ^1^O_2_ through its near-infrared phosphorescence, or O_2_^•−^ through electronic paramagnetic resonance [11,12], or when the analyte reacts with some chemical probe which is then detected by suitable analytical techniques [13]. Direct techniques, while providing the advantage of specificity, lack in most cases the required sensitivity and require costly specialized equipment, which makes the indirect approach more convenient and widespread, especially when conventional techniques, such as absorption or fluorescence spectroscopy, are used [14,15]. Several selective fluorescent probes for the detection of the most important ROS/RNS have been extensively developed and their use has been protocolized [16]. However, these optical techniques also present severe drawbacks when applied to biological systems, let alone living organisms, the main one is the poor penetration of light, e.g., in human tissue, and the strong scattering that prevent the detection of light emitted or reflected by deep targets [17]. These limitations can be circumvented using photoacoustic techniques, which provide deep tissue penetration [18,19]. For example, different internal organs such as the liver, lung, or brain have been imaged in vivo using photoacoustic techniques. 

The photoacoustic phenomenon was discovered by A.G. Bell, more than one century ago [20]. Briefly, when a molecule absorbs radiant energy, an excited state is created that eventually releases the acquired extra energy by a combination of radiative and non-radiative decay processes. In some cases, it may undergo also a photochemical reaction. Non-radiative decay processes cause a local heating of the molecules’ microenvironment and hence its thermal expansion, leading to a pressure wave that propagates through the medium. This pressure wave can be detected by an ultrasonic microphone (e.g., a piezoelectric detector). The intensity of the photoacoustic signal is proportional to the light-induced pressure change, i.e., to the amount of heat released. Additionally, light-induced molecular rearrangements can also result in volume changes and may also contribute to the photoacoustic signal. The main advantage of detecting ultrasound waves in comparison with visible light is that their propagation is less affected by absorption and/or cell scattering processes (or the tissue in vivo) [21,22].

Photoacoustic imaging relies on the use of photoacoustic probes, substances that, after interaction with an analyte, change their ability to induce a pressure wave upon exposure to light. An ideal photoacoustic probe should fulfill a series of requirements: i) it should have a high molar absorption coefficient, ii) it should release all the absorbed energy as heat as fast as possible, and iii) it should have high photobleaching resistance [23]. As photoacoustic probes are comparatively less developed than fluorescent probes, early works on biological photoacoustic imaging used the available fluorescent probes [24,25,26,27,28]. In 2014, the first ROS photoacoustic nanosensor was developed by embedding a cyanine 7 derivative to a semiconductive polymeric nanoparticle [29]. Since then, a few more examples of photoacoustic nanosensors have been developed for ROS/RNS detection (e.g., a photoactivatable, reversible or theranostic photoacoustic nanosensor among others) [30,31,32].

The use of fluorescent probes for photoacoustic imaging seems far from efficient because fluorescent dyes will waste a fraction of their excited-state energy by emitting a photon. Hatamimoslehabadi et al. have compared the photophysical properties with the photoacoustic response for a selection of established chromophores, revealing the importance of a large non-radiative/radiative decay rate constant ratio for a good photoacoustic performance [33]. For example, fluorescein is a highly emissive dye with poor photoacoustic properties, while phenolphthalein, a fluorescein derivative, is non-fluorescent yet shows good photoacoustic properties [34]. Weakly fluorescent dyes or intrinsic fluorescent nanomaterials (e.g., nanodiamonds) have been developed and used as dual (fluorescence and photoacoustic) imaging agents [35,36]. Thus, fluorescent dyes with small fluorescent quantum yield have been used for photoacoustic imaging in the red or near-infrared regions [37]. More complex strategies such as fluorophore aggregation or non-linear absorption processes have also been used to boost the photoacoustic signal [38,39].

Recently, a simple approach has been proposed that boosts the responsivity of photoacoustic probes, namely, the use of acoustogenic dyes that change color upon reaction with the analyte of choice [40]. The acoustogenic dye is photoacoustically silent in its native form and produces a photoacoustic signal after interaction with the analyte, offering, in principle, infinite contrast. We performed a bibliographic search to identify potential acoustogenic dyes highly reactive against ROS/RNS. A non-colored, non-toxic, and non-carcinogenic compound that reacts with some ROS generating a blue oxidized complex is 3,3′,5,5′-tetramethylbenzidine (TMB) [41,42,43]. TMB is currently used as a visualizing reagent in ELISA assays, taking advantage of its oxidation with H_2_O_2_ in the presence of horseradish peroxidase [44]. 

**Scheme 1:**
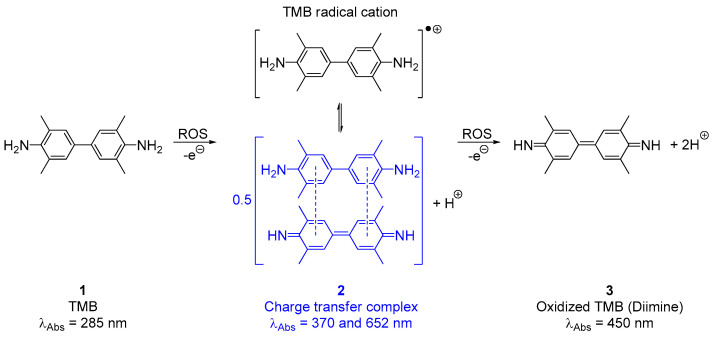
TMB (3,3′,5,5′-tetramethylbenzidine) reactivity towards oxidant agents.

As shown in Scheme 1, TMB reacts with an oxidant agent to yield a radical cation (TMB^•+^), which forms a blue (λ_abs_ = 652 nm) charge transfer complex (**2**) with a second TMB molecule. In some cases, the presence of an additional oxidizing agent leads to a diimine product (**3**), which has a yellow coloration (λ_abs_ = 450 nm) [41,43,45]. Finally, with a large excess of an oxidizing agent, the reaction will proceed to yield other non-colored oxidized derivatives. In this work, we have assessed the potential of TMB as an acoustogenic photoacoustic probe for ROS/RNS using laser-induced optoacoustic spectroscopy (LIOAS). We have studied its reactivity towards different ROS/RNS in water and have used it to detect ^1^O_2_ generated in *E. coli* cells.

## 2. Materials and Methods

### 2.1. Materials

TMB (3,3′,5,5′-tetramethylbenzidine), sodium hypochlorite solution (50 g/l in H_2_O; NaClO), potassium superoxide (KO_2_), Rose Bengal (RB), 2,2,6,6-tetramethylpiperidin-1-yl)oxyl (TEMPO), sodium azide (NaN_3_), bromocresol purple (BCP) and phosphate-buffered saline (PBS; pH = 7.4) were purchased from Sigma-Aldrich (St. Louis, MO, USA). Hydrogen peroxide solution (30 wt % in H_2_O; H_2_O_2_), sodium nitrite (NaNO_2_), sodium nitroprusside (Na_2_[Fe(CN)_5_NO]), and Luria–Bertani (LB) medium were supplied by Panreac (Barcelona, Spain). MDPyTMPyP (5-mono(N-decyl-4-pyridyl)-10,15,20-tri(N-methyl-4-pyridyl)-21H,23H-porphine tetrachloride) was supplied by Frontier Scientific (Logan, UT, USA). All reagents were used as received.

### 2.2. General Spectroscopic Measurements

All spectroscopic measurements were carried out in 1-cm quartz cuvettes (Hellma, Germany) at room temperature. Absorption spectra were recorded on a Cary 6000i spectrophotometer (Varian, Palo Alto, CA, USA).

The time-resolved photoacoustic signals were recorded using home-built LIOAS equipment. TMB was excited using a Q-switched Nd:YAG laser (Surelite I-10, Continuum, CA, USA) coupled to an optical parametric oscillator (OPO) laser (SL OPO, 5 ns pulse width, 10–100 μJ per pulse, Continuum, CA, USA). The excitation wavelengths were 652 nm or 450 nm depending on the experiment. The laser power was varied using neutral density filters. The laser beam was passed through a vertical slit of dimensions 5 × 1 mm immediately before entering the cuvette. Then, the laser-induced pressure changes in the sample were detected by a Panametrics piezoelectric transducer pressed to the sidewall of a cuvette and held in a temperature-controlled holder (Quantum Northwest, WA, USA). A thin layer of silicon grease (Baysilone-35, Bayer, Leverkusen, Germany) ensured optimum acoustic coupling between the cuvette and the piezoelectric transducer. After amplification, the signal was fed to a Lecroy Wavesurfer 454 oscilloscope (Teledyne LeCroy, VA, USA) for digitizing and averaging (typically 1000 shots) and finally transferred to a PC for data storage and analysis. The amplitude of photoacoustic signal maximum (*H*_max_) is proportional to the absorbed laser energy, and therefore to the energy of the laser pulse (*E*) and the sample absorption factor by (1).

(1) $$H_{max}\;=\;k^{\prime }\emptyset \left[1\;-\;10^{-A}\right]$$

where *k’* is a proportional constant that accounts for electronic and geometric factors of the experimental system and *ϕ* is the *photoacoustic efficiency* [46]. In optically thin samples, the absorption factor is proportional to the concentration of the absorber (c), which can be taken advantage of for absorber quantification purposes. Thus, Equation (1) can be simplified to Equation (2):

(2) $$H_{max}\;\sim \;k^{\prime \prime }\emptyset c$$

The *ϕ* value is determined by comparing *H*_max_ for optically matched solutions of the sample and a suitable photoacoustic reference (BCP dissolved in 0.1 M NaOH, with *ϕ* = 1, was used in this work [47]) as indicated in Equation (3).

(3) $$\emptyset_{Sample}=\;\frac{H_{max;Sample}}{H_{max;Ref}}\emptyset_{Ref}$$

### 2.3. Statistical Analysis

The limit of detection (LOD) and the limit of quantification (LOQ) were calculated as 3- and 10-fold the standard deviation of the blank interpolated to the analyte calibration curve. The standard deviation of the blank was obtained from ten independent blank samples.

### 2.4. Sources of the ROS and RNS Tested

The different ROS and RNS used in this work were prepared according to the protocols below. Sodium hypochlorite (NaOCl) was diluted from the commercial source (50 g/L of NaClO in water). Superoxide anion radical (O_2_^•−^) was added as solid KO_2_. Hydrogen peroxide (H_2_O_2_) was diluted from the commercial source (H_2_O_2_ 30% in water). Hydroxyl radical (^•^OH) was generated by irradiation of 10 mM NaNO_2_ solution with UV-A light (354 ± 20 nm) [48]. The generation of ^•^OH was verified by scavenging it with terephthalic acid and mannitol (Figure S1; Schemes S1 and S2) [49]. Singlet oxygen (^1^O_2_) was generated by irradiation of Rose Bengal (a well-known ^1^O_2_ photosensitizer) [50] with green light (520 ± 18 nm). Nitric oxide (^•^NO) was generated from the thermal decomposition of an aqueous solution of 1 M sodium nitroprusside (Na_2_[Fe(CN)_5_NO]) [51]. Peroxynitrite anion (ONOO^−^) was generated as described in [52]. Briefly, 1.5 M NaOH (aq) was added to a mixture of 0.6 M NaNO_2_, 0.7 M H_2_O_2,_ and 0.6 M HCl. The ONOO^−^ solution was used immediately after its preparation. Nitrogen dioxide (^•^NO_2_) was generated from the decomposition of sodium nitrite (NaNO_2_) [53]. Briefly, 1 M NaNO_2_ solution was added to H_2_SO_4_ (96%) and the generated brownish gas was collected in a balloon and injected into a sealed cuvette containing the TMB solution. Nitrite (NO_2_^−^) was diluted from a 1 M sodium nitrite solution. Then, 2,2,6,6-tetramethylpiperidin-1-yl)oxyl (TEMPO) was added as solid.

### 2.5. Bacterial Cell Cultures

#### 2.5.1. *Escherichia coli* Cells with the Endogenous PS MiniSOGs 

*E. coli* cells (DH10β, Invitrogen, CA, USA) were transformed with the expression vector encoding the fluorescent proteins miniSOG wt, miniSOG Q103L, and miniSOG Q103V. Cells were grown aerobically from a single colony to an OD_600_ = 0.3 in Luria–Bertani (LB) medium supplemented with 100 µg/mL ampicillin at 37 °C. Untransformed DH10β cells were grown in LB without ampicillin. Protein expression was induced by the addition of 0.2% (w/v) *L*-arabinose. Cells were harvested by centrifugation after 3 h. Afterward, the cells were incubated with TMB (200 μM) for 10 min and washed three times with PBS (pH 7.4). 

#### 2.5.2. *Escherichia coli* Cells with the Exogenous PS MDPyTMPyP

*E. coli* cells (ATCC 25922, Manassas, VA, USA) were grown aerobically from a single colony to an OD_600_ = 0.3 in LB medium at 37 °C. Then, cells were harvested by centrifugation and washed three times with PBS (pH 7.4). Afterward, cells were incubated with MDPyTMPyP (10 μM), TMB (200 μM), and NaN_3_ (if it was necessary; 50 mM) for 10 min, followed by three washes with PBS (pH 7.4).

## 3. Results

### 3.1. Photoacoustic and Spectroscopic Properties of TMB and Its Oxidation Products (2 and 3)

**Figure 1:**
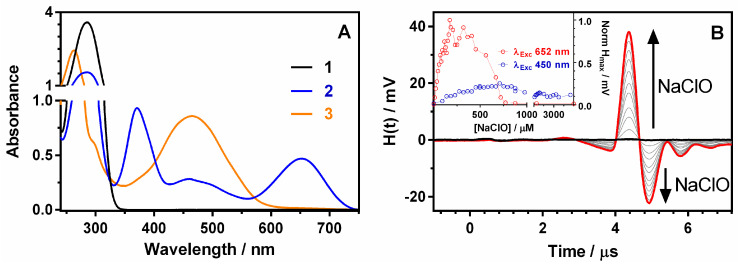
(**A**): Absorption spectra of TMB, **2** and **3,** in PBS. (**B**): TMB photoacoustic waveforms enhancement upon successive NaClO additions in PBS ([TMB] = 200 μM; [NaClO] = 0–150 μM;λ_exc_ = 652 nm). Inset: Photoacoustic maximum amplitude in function of the amount of NaClO added. The excitation wavelengths were 652 (**2**; red line) and 450 (**3**; blue line) nm. [TMB] = 200 μM.

The absorption spectra of TMB, the blue complex **2**, and the yellow diimine **3** in PBS are shown in Figure 1A. TMB absorbs only in the ultraviolet region with a band centered at 290 nm. Upon addition of NaClO, we observed the formation of the blue complex **2**, which absorbs throughout the visible spectrum with bands at 650, 460, and 370 nm. When a light excess of NaClO relative to TMB was added, the blue complex **2** evolved to the yellow diimine **3**, which shows a single absorption band at 450 nm. Further addition of NaClO oxidized **3** to uncolored products.

Based on the absorption spectra of TMB, **2,** and **3**, we chose 652 nm for probing **2** and 450 nm for probing **3** in the photoacoustic experiments. A clear photoacoustic signal at 652 nm appeared upon the addition of NaClO, which we ascribe to the formation of **2** (Figure 1B). The amplitude of the photoacoustic signal increased up to a maximum and then began to decrease, indicating the disappearance of **2**. Two factors contributed to this: as oxidation proceeds, there is less and less TMB available to form the complex **2**, resulting in the saturation of the signal at NaClO concentration ~ 250 μM. Besides, TMB^•+^ is further oxidized to the diamine **3**, which was demonstrated by the concomitant growth of the photoacoustic signal at 450 nm and its decrease at 652 nm at high NaClO concentration (Figure 1B inset).

### 3.2. Factors Affecting the Photoacoustic Signal

**Figure 2:**
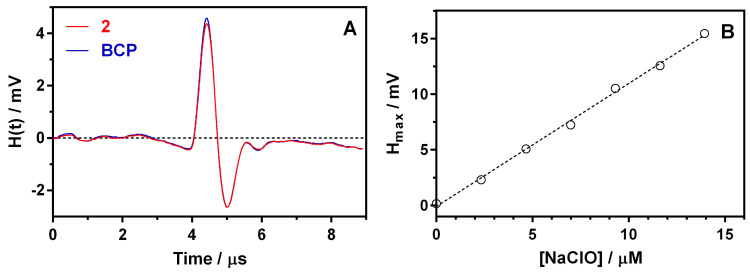
Determination of *ϕ* for **2** (red line) in PBS (pH 7.4). The reference photoacoustic wave was obtained using an optically matched bromocresol purple solution (BCP; *ϕ* = 1 (0.1 M NaOH); blue line) [46] at the excitation wavelength (λ_Exc_ = 652 nm). (**B**): Photoacoustic maximum amplitude vs. concentration of NaClO added in PBS (pH 7.4). [TMB] = 200 μM; λ_exc_ = 652 nm.

The amplitude of the photoacoustic signal reflects the amount of heat deposited in the system through radiationless decay of the excited states, as well as the differences in molecular volumes between the excited- and the ground-state species. Comparing the amplitude of the photoacoustic signal for **2** with that of the photocalorimetric reference BCP, the photoacoustic efficiency of **2** was determined as *ϕ* = 0.95 ± 0.10 (Figure 2A). Kinetic analysis of the time-resolved photoacoustic signal revealed that the excited state decay of **2** is faster than the temporal resolution of the technique. Finally, **2** was found to be non-fluorescent. These results indicate that **2** is an ideal photoacoustic probe since it releases fast and efficiently as heat all the absorbed light energy. Additionally, the photoacoustic signal increases linearly with the excitation laser power (Figure S2) and is fairly photostable, since 8500 laser shots of 100 μJ are needed to reduce the signal by half (Figure S3), while to obtain a decent signal 10 shots of 10 μJ suffice. 

Next, the effect of the TMB concentration was examined. The highest photoacoustic amplitude was observed when two equivalents of TMB reacted with one equivalent of NaClO, therefore this proportion was kept for all experiments. A linear increase in the photoacoustic signal amplitude with TMB was observed up to roughly 30 μM and the signal leveled off around 200 μM (Figure S4) in agreement with Equation (1). Monitoring of **2** enables measurements of NaClO concentration up to 100 μM. *H*_max_ increases linearly with NaClO up to a concentration of 15 μM (Figure 2B). The precision of the method has been assessed using 10 independent replicates obtaining a standard deviation of 15% (Figure S5), which is in line with typical standard deviation values for LIOAS. The detection and quantification limits have then been estimated as 0.6 and 1.4 μM, respectively (Figure S6). 

### 3.3. TMB Reactivity Towards the Tested ROS, RNS, and TEMPO

**Figure 3:**
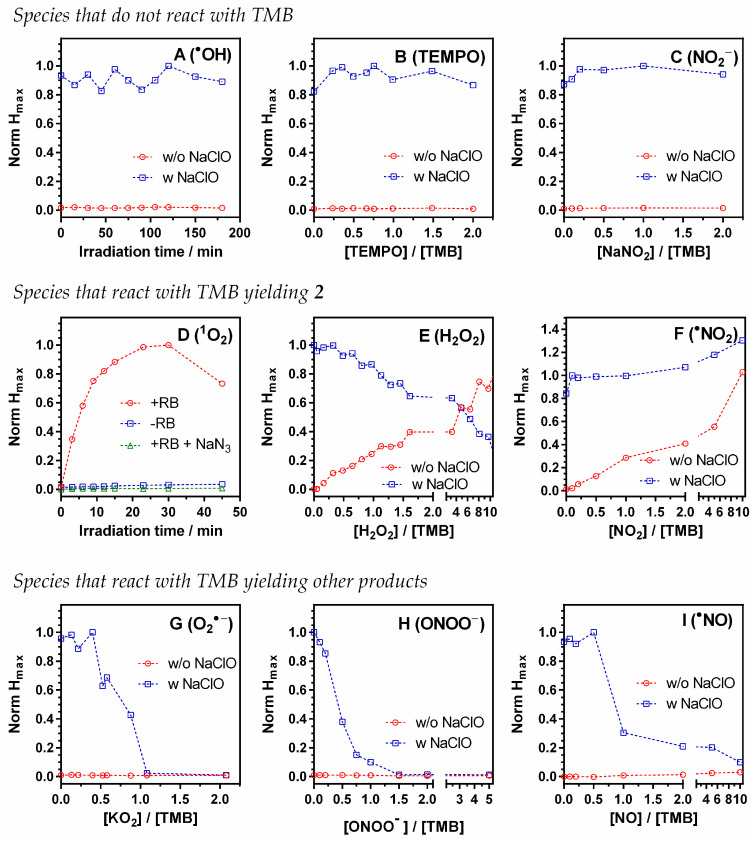
Photoacoustic maximum amplitude of TMB after reacting with different ROS and RNS. (**A**): Hydroxyl radical (^•^OH), generated via sodium nitrite photolysis in water (λ_exc_ = 354 ± 20 nm). (**B**): (2,2,6,6-Tetramethylpiperidin-1-yl)oxyl (TEMPO). (**C**): Sodium nitrite (NaNO_2_). (**D**): Singlet oxygen (^1^O_2_), generated via Rose Bengal irradiation (λ_exc_ = 520 ± 18 nm). (**E**): Hydrogen peroxide (H_2_O_2_). (**F**): Nitrogen dioxide (^•^NO_2_), generated via sodium nitrite acid decomposition. (**G**): Superoxide radical anion (O_2_^•−^). (**H**): Peroxynitrite (ONOO^−^). (**I**): Nitric oxide (^•^NO), generated via sodium nitroprusside decomposition. As a control for B, C, E, F, G, H after the acquisition of the photoacoustic signal, 100 μM NaClO was added and their photoacoustic signal was measured again (red and blue line, respectively).

**Scheme 2:**
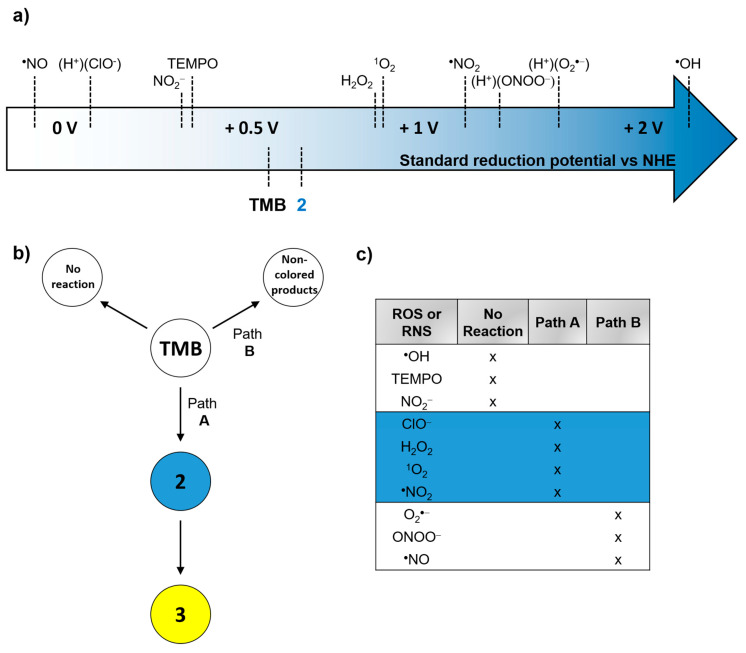
(**a**) Standard reduction potentials for the different tested ROS, RNS, TEMPO, and TMB [55,56,57,58,62]. (**b**) The possible reaction pathways of TMB towards different ROS or RNS. (**c**) Table summarizing the reactivity towards TMB of the different ROS and RNS studied.

After establishing the spectroscopic and photoacoustic properties of TMB, **2,** and **3**, we determined the reactivity of TMB towards other ROS, RNS, and the stable free radical TEMPO (Figure 3). Thus, TMB was allowed to react with them for 5 min before the photoacoustic response was recorded. For TEMPO and the stable ROS/RNS, we recorded the photoacoustic response at oxidant:TMB ratios ranging from 1:20 up to 10:1 eq, whereas for oxidants generated photochemically, we evaluated the photoacoustic response as a function of the irradiation time. When a species would not show any photoacoustic signal, we repeated the experiment in the presence of 100 μM ClO^−^ as a control. The results are summarized in Scheme 2. Three types of response were observed:

#### 3.3.1. Species That Give no Photoacoustic Signal

This was the case for ^•^OH, TEMPO, and NaNO_2_ (Figure 3, panels A, B, C). TEMPO and NaNO_2_ did not react with TMB under these conditions. It is well known that nitrous acid HNO_2_ reacts with aromatic amines to form diazonium salts and leading eventually to the formation of colored diazo compounds. However, the p*K*_a_ of HNO_2_ is 3.15, therefore this reaction is not expected to occur at physiological pH [54]. The reduction potential of nitrite is 0.375 V vs. NHE [55], while the reduction potential of the **2** is +0.553 V [56], therefore production of **2** is not favorable. TEMPO has a very similar reduction potential (+0.380 V) [57], therefore no reaction is to be expected either. On the other hand, TEMPO may also react abstracting a hydrogen atom from TMB, however, the extent of this reaction seems to be negligible under our experimental conditions. Surprisingly, ^•^OH does not react with TMB despite having a most favorable reduction potential [58]. In general, ^•^OH reactions tend to be diffusion-controlled owing to their strong reactivity. Indeed, ^•^OH reacts with TPA at the same concentration used for TMB (200 μM; Figure S1). As a final control, we added 100 μM ClO^−^ to the analyzed solution and repeated the measurement. Reassuringly, the extent of production of **2** was the same in the absence and the presence of ^•^OH. The same holds for TEMPO and NaNO_2_.

#### 3.3.2. Species That React with TMB Yielding 2

In addition to ClO^−^ (Figure 1), **2** was formed in the presence of H_2_O_2_ (+0.80 V), ^1^O_2_ (+0.81 V), and ^•^NO_2_ (+1.04 V), which have sufficiently high reduction potentials for oxidizing TMB (Figure 3, panels D, E, F) [58]. The photoacoustic signal increases in an almost linear fashion with the concentration of H_2_O_2_, although the maximum amplitude is not reached until an H_2_O_2_:TMB ratio of about 15. This most likely indicates that the reaction with TMB is slow. Bearing in mind the prospective use of TMB for measurements in biological samples, we ruled out extending the time lag between TMB addition and measurement as well as the addition of accelerators such as horseradish peroxidase. The addition of 100 μM ClO^−^ to the analyzed solutions produced a signal that decreased with the concentration of H_2_O_2_ in a complementary fashion to the increase observed in its absence. The sum of the signals before and after the addition of ClO^−^ is approximately 100% in all cases. This indicates that ClO^−^ oxidizes the TMB that did not react H_2_O_2_, adding to the signal. Alternative reactions between ClO^−^ and H_2_O_2_, e.g., the production of ^1^O_2_ [59] cannot be ruled out. 

Figure 3D shows that ^1^O_2_ reacts readily with TMB to produce the blue complex **2**. ^1^O_2_ is a metastable ROS that has a lifetime of 3.5 μs in PBS. Therefore, it was generated in situ by irradiation of Rose Bengal (a well-known ^1^O_2_ photosensitizer) with green light [50]. The amount of ^1^O_2_ generated is proportional to the irradiation time. The photoacoustic signal shows a ^1^O_2_ dependence very similar to that of ClO^−^, namely, a linear growth at short irradiation times (i.e., when the number of ^1^O_2_ molecules generated is small, relative to that of TMB molecules), followed by saturation at longer times, corresponding to the maximum concentration of the complex **2**, and eventually to a steady signal decrease, indicating a shortage of reduced TMB molecules to sustain the complex **2**, as well as further oxidation of **2**. When the experiment was repeated in the absence of RB or the presence of added 10 mM sodium azide, a well-known ^1^O_2_ scavenger [60], no photoacoustic signal could be observed beyond the base level. Thus, the formation of the blue complex **2** was indeed due to the reaction of TMB with photogenerated ^1^O_2_.

The final oxidant in this group, ^•^NO_2_, shows a behavior very similar to that of H_2_O_2_ (Figure 3F). It reacts with TMB to yield the blue complex **2** and the reaction is not yet complete when the photoacoustic signal is recorded unless ^•^NO_2_ is added in 10-fold excess. Unlike H_2_O_2_, the addition of ClO^−^ gives the 100% signal system and even rises above this level at the highest ^•^NO_2_ concentrations, most likely due to light absorption by ^•^NO_2_ itself (σ_650_ = 1.54 × 10^−20^ cm^2^/molecule; gas phase) [61].

#### 3.3.3. Species That React with TMB Yielding Non-Colored Products

This was the case of O_2_^•−^, ONOO^−^ and ^•^NO, which did not form the blue charge-transfer complex **2** at any concentration or produced only minute amounts of it (Figure 3 panels G, H, I). As in the case of ^•^OH, this was surprising because the reduction potentials are very favorable (+1.46 V for O_2_^•−^ and +1.20 V for ONOO^−^); less so for ^•^NO, −0.15 V [58,62]. Unlike the observations made for ^••^OH, the subsequent addition of NaClO did not produce the 100% photoacoustic signal but a signal that decreased with the initially added concentration of the ROS/RNS as in the H_2_O_2_ case. This indicates that TMB had been consumed, however, yielded products different than **2**. Since our goal was to assess the usefulness of TMB as a photoacoustic probe for ROS/RNS, we did not seek to investigate the nature of such products. Nevertheless, a possible explanation for the strong oxidants O_2_^•−^ and ONOO^−^ is that TMB was oxidized to the diamine **3.** Besides, it has been reported that O_2_^•−^ can oxidize aniline (taken as a simple TMB model) to their correspondent nitro or nitroso derivatives [63] as well as to 4-nitro-*N*-phenylaniline [64]. Similarly, ONOO^−^ and ^•^NO can react with primary and secondary amines to yield *N*-nitrosamines and *N*-nitramines [65]. As a final note, it is worth noting, that for a high concentration of ^•^NO (20- and 30-eq.) a small amount of TMB is oxidized to yield **2**, confirming the minor contribution of the electron-transfer reaction pathway.

### 3.4. Detection of ROS in Biological Media

**Figure 4:**
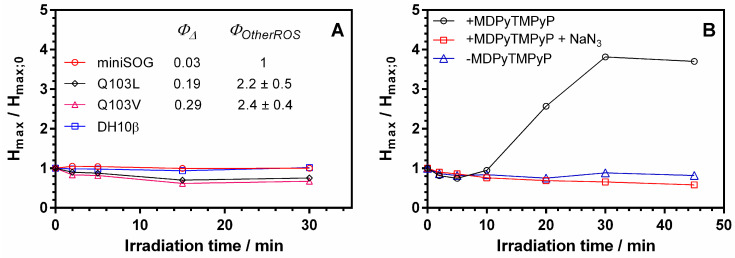
Photoacoustic maximum amplitude enhancement of TMB for (**A**) untransformed DH10β (blue squares) and miniSOG-expressing (red circles, black rhombuses, and magenta triangles for miniSOG, miniSOG Q103L and miniSOG Q103V, respectively) *E. coli* cells for different irradiation times (lamp power 14.0 mW/cm^2^; λ_Irr_ = 459 ± 10 nm). The values of ^1^O_2_ quantum yield (*Φ_Δ_*) and relative generation of other ROS (*Φ_OtherROS_*) by miniSOG and its mutants Q103L and Q103V are taken from [68]. (**B**): *E. coli* ATCC 25922 cells in the presence (200 μM; black circles) and absence (blue triangles) of MDPyTMPyP for different irradiation times (lamp power 5.5 mW/cm^2^; λ_Irr_ = 420 ± 20 nm). The control experiment was realized coincubating MDPyTMPyP with 50 mM NaN_3_ (red squares).

In the previous section, we established that TMB is a useful photoacoustic probe for ClO^−^ and ^1^O_2_ and, to a lesser extent, also for H_2_O_2_ and ^•^NO_2_. We then sought to assess its performance in biological media. To this end, we added TMB to suspensions of wt DH10β *E. coli* bacteria as well as three genetically modified strains that express the ROS photosensitizing proteins miniSOG and its mutants Q103V and Q103L, respectively. These are flavin-binding proteins that produce ^1^O_2_ and other ROS in different proportions under exposure to blue light (460 nm) [66,67,68]. All strains produced a photoacoustic signal at 652 nm, likely due to light absorption by heme. However, the signal was not enhanced upon exposure of the bacteria to blue light (Figure 4A and Figure S7). This indicates that no blue complex is formed, which may be due to TMB and the ROS being in distant cellular locations. Since the photosensitizing proteins are located in the cytoplasm, the most likely explanation is that TMB is not internalized by the bacteria. 

To confirm this, we conducted a second experiment in which the ATCC 25922 *E. coli* cells were incubated with the porphyrin MDPyTMPyP (Scheme S3). We had previously demonstrated that the porphyrin MDPyTMPyP accumulates in the bacterial outer membrane and produces the characteristic ^1^O_2_ emission at 1270 nm under exposure to light [69]. In this photoacoustic experiment, a clear enhancement was observed upon irradiation with 420 nm blue light, confirming the formation of the blue complex **2** (black line in Figure 4B and Figure S8). No enhancement was observed in the absence of MDPyTMPyP (blue line in Figure 4B). Next, we addressed the question of whether TMB binds to the bacteria or remains in the bulk solution. When we centrifuged the suspension and recorded the photoacoustic signal of the supernatant solution, no signal could be observed. However, we could recover it by resuspending the bacterial pellet (Figure S9). This indicates that TMB binds strongly to bacterial cells, but is not internalized by them. As a final experiment, we observed that the addition of 50 mM NaN_3_ (a selective ^1^O_2_ scavenger) prevented the growth of the signal (red line in Figure 4B) [60]. Taken together, these experiments indicate that TMB can detect ^1^O_2_ in biological media.

Closer inspection of the time evolution of the photoacoustic signal reveals a time lag between the beginning of irradiation and the onset of the signal. This behavior has been observed also for fluorescent ROS probes in cells and is believed to reflect the scavenging of the first waves of ^1^O_2_ by cellular antioxidants [70]. When these are consumed, TMB oxidation proceeds.

### 3.5. Discussion

Photoacoustic imaging is based on the radiationless deactivation of photoexcited probes, a universal process. Photoacoustic probes have some advantages over fluorescent probes, such as the better transmission of sound in highly scattering biological media, and the possibility of tissue-depth profiling derived from the slower velocity of sound propagation [18]. In addition to these, acoustogenic probes offer the added advantage of, potentially, infinite contrast between the signals of the native and oxidized forms, derived from the color change [40]. This work shows that TMB is an acoustogenic photoacoustic probe that reacts with several ROS/RNS to yield a blue complex **2**, which shows a photoacoustic signal with efficiency close to 100% when probed at 652 nm. The main properties of TMB are summarized in Table 1.

ROS/RNS probes can be classified as specific or non-specific. Selectivity is useful to pinpoint the role of a specific species in a chemical or biological process, as well as to quantify its concentration. However, in biological media, ROS interconvert very often through a complex network of pathways [1], therefore the use of specific probes is less critical. Non-specific probes such as 2′,7′-dichlorodihydrofluorescein are of widespread use in fluorescence microscopy as they provide useful information on overall oxidative stress [71]. We anticipate a similar role for TMB, which does not detract from the interest in developing specific acoustogenic probes for particular ROS/RNS.

As a final comment, the ability to report the ROS level in biological media is an essential feature of probes, e.g., for dosimetry purposes in oxidation-based therapies such as photodynamic therapy [72,73]. Our work successfully demonstrates that TMB does not penetrate bacterial cells but is capable of detecting ^1^O_2_ produced in the outer membrane of *E. coli.* There is little doubt that, as the photoacoustic imaging field will develop, other probes will be able to do this. Nevertheless, one of the most popular singlet oxygen fluorescent probes, Singlet Oxygen Sensor Green, is marketed as cell impermeant [74] and does not penetrate *E. coli* cells either [75].

## 4. Conclusions

Photoacoustic probes circumvent the main limitations of fluorescent probes, particularly tissue penetration and the need for transparent media. Besides, they can report in a depth-resolved manner taking advantage of the lower speed of propagation compared to light. We have demonstrated that the chromogenic TMB is a suitable photoacoustic probe for red-light monitoring of ClO^−^, ^1^O_2,_ and, to a lesser extent, H_2_O_2_ and ^•^NO_2_ due to the formation of a blue complex upon oxidation. Other ROS/RNS either do not react with TMB at any appreciable rate (^•^OH, NO_2_^−^, and the TEMPO radical) or yield photoacoustically silent products (O_2_^•−^, ONOO^−^, and ^•^NO). TMB binds to the outer membrane of the *E. coli* bacterial cell wall and aptly reports on ^1^O_2_ located in its vicinity. The results of this work may pave the way to the development of new probes for photoacoustic imaging of a wide variety of biologically-relevant species, thereby providing a sensitive and selective alternative to fluorescence imaging.

## Figures and Tables

**Table 1:** Main relevant properties of TMB (or their oxidation products).

Type of Probe	Acoustogenic
*λ*_max_ / nm	652
*ϕ ^a^*	0.95 ± 0.10
LOD *^b^* (ClO^−^) / μM	0.6
LOQ *^c^* (ClO^−^) / μM	1.4
Responsivity	ClO^−^, ^1^O_2_, H_2_O_2_, ^•^NO_2_

*^a^* Photoacoustic efficiency. *^b^* Limit of detection. *^c^* Limit of quantification.

## Supplementary Materials

The following are available online at https://www.mdpi.com/1424-8220/20/20/5952/s1, Figure S1: Reactivity of terephthalic acid towards ^•^OH. Figure S2: Laser energy dependence of photoacoustic maximum amplitude for **2**. Figure S3: Photostability of **2** upon 652 nm laser-pulsed irradiation. Figure S4: Photoacoustic maximum amplitude for **2** as a function of the initial TMB concentration upon reaction with ClO^−^. Figure S5: Precision test. Figure S6: Limit of detection and limit of quantification test. Figure S7: Photoacoustic waveforms for different *E. coli* cell-suspension encoding different ROS generating proteins (miniSOG, miniSOG Q103L, miniSOG Q103V) as a function of the irradiation time. Figure S8: Photoacoustic waveforms for *E. coli* cell-suspension incubated with or without 10 μM MDPyTMPyP as a function of the irradiation time. Figure S9: Photoacoustic waveforms for the supernatant and the pellet resuspension for *E. coli* cell-suspension incubated with 10 μM MDPuTMPyP and irradiated for 45 min. Scheme S1: Reactivity of terephthalic acid towards ^•^OH. Scheme S2: Photolysis of NaNO_2_ in an aqueous environment to generate ^•^OH. Scheme S3: Chemical structure of MDPyTMPyP.
