# Supplementary material for: Tetramethylbenzidine: An Acoustogenic Photoacoustic Probe for Reactive Oxygen Species Detection
Source: Sensors (Basel). 2020 Oct 21;20(20):5952. doi: 10.3390/s20205952 (PMC7590141; doi:10.3390/s20205952)
Supplement: Supplementary file 1 [file sensors-20-05952-s001.pdf]

Supporting Information

Tetramethylbenzidine: a photoacoustic probe for reactive oxygen species detection

Roger Bresolí-Obach [1,2], Marcello Frattini [1,3], Steffania Abruzetti [3,4], Cristiano Viappiani [3,4], Montserrat Agut [1] and Santi Nonell [1,*]

1 Institut Químic de Sarrià, Universitat Ramon Llull, Via Augusta 390, 08017 Barcelona, Spain. roger.bresoliobach@kuleuven.be, mfrattini7@gmail.com, montserrat.agut@iqs.url.edu, santi.nonell@iqs.url.edu

2 Current address: Department of Chemistry, Katholieke Universiteit Leuven, celestijnenlaan 200F, 3001 Leuven, Belgium.

3 Dipartimento di Scienze Matematiche, Fisiche e Informatiche, Università di Parma, Parco Area delle Scienze 7A, 43124 Parma, Italy. stefania.abbruzzetti@unipr.it, cristiano.viappiani@unipr.it

4 NEST, Istituto Nanoscienze, Consiglio Nazionale delle Ricerche, Piazza San Silvestro 12, 56127 Pisa, Italy.

* Correspondence: santi.nonell@iqs.url.edu; Tel.: +34-93-267-2000

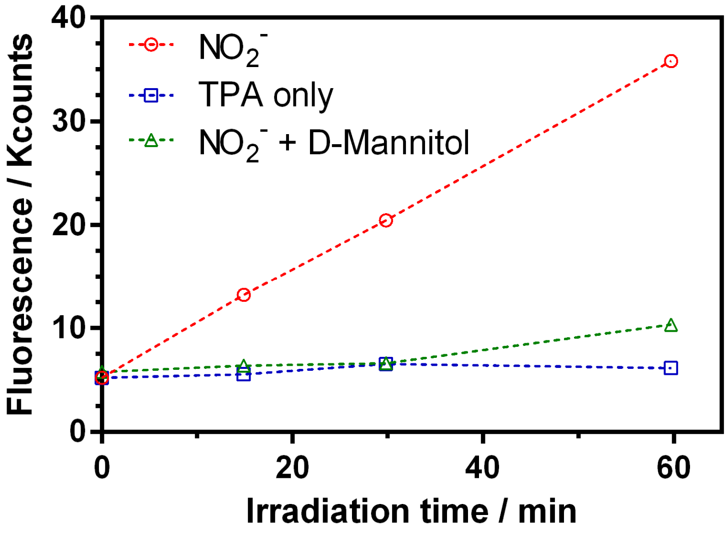

Figure S1. Terephthalic acid (TPA; λ_{exc} = 312 nm; λ_{det} = 410-500 nm) fluorescence increase upon photolysis of 10 mM $NaNO_2$ under UV-A irradiation (red line; 354 ± 20 nm; for further details about the photogeneration of •OH see Scheme S1 and S2). In addition, this experiment has also been recorded in presence of 60 mM D-mannitol (a well-known hydroxyl scavenger;[1] green line) and without the addition of $NaNO_2$ (blue line) as controls. In this experiment we used TPA as •OH fluorescent probe.[2]

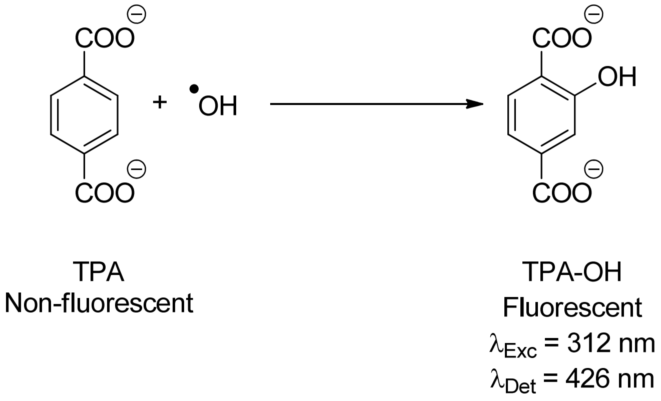

Scheme S1. •OH reacts with TPA to yield a highly-fluorescent mono-hydroxylated product (TPA-OH).[2]

$$NO_2^{\ominus} + H_2O \xrightarrow{h\nu\ (>300\ nm)} NO + {}^{\bullet}OH + OH^{\ominus}$$

Scheme S2. Photolysis of $NaNO_2$ in an aqueous environment to generate •OH.[3]

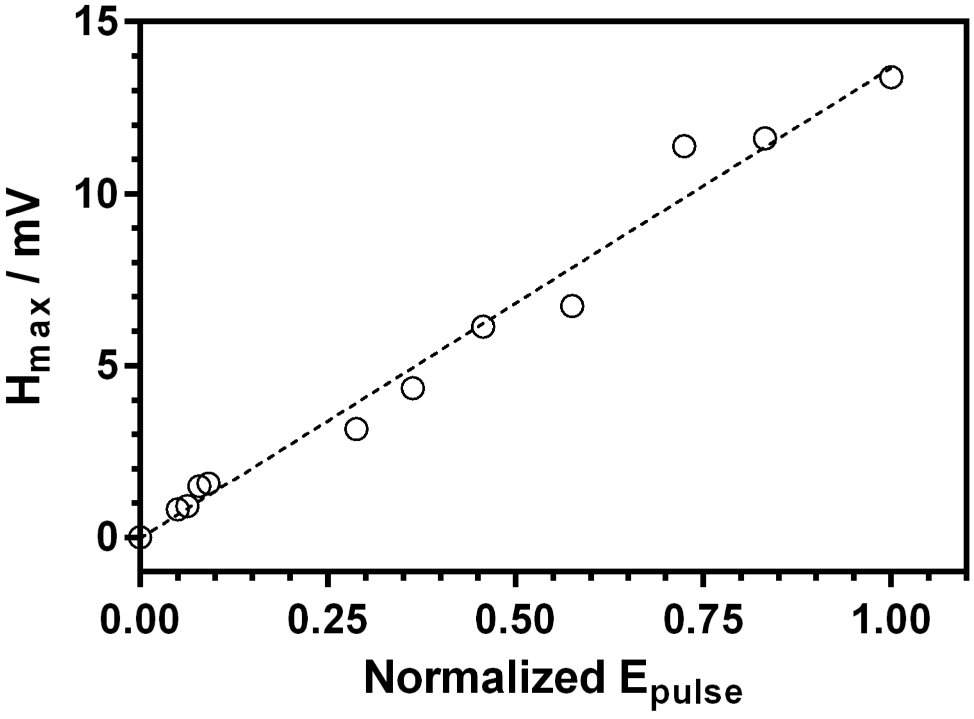

Figure S2. Laser energy dependence of photoacoustic maximum amplitude for **2**. λ_{exc} = 652 nm.

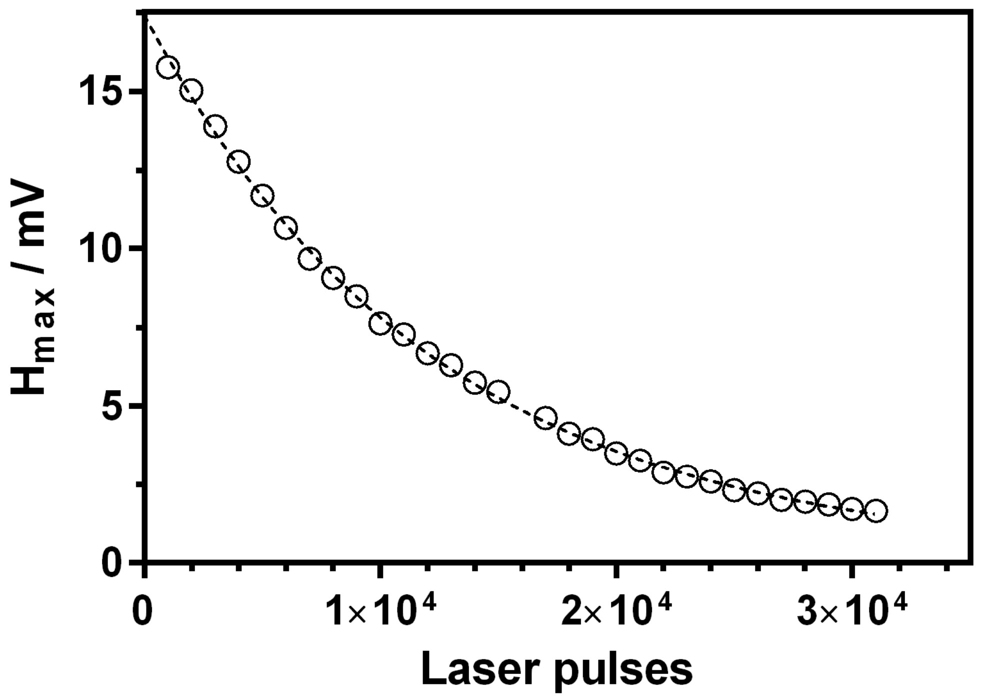

Figure S3. Photostability of **2** upon 652 nm laser-pulsed irradiation (E_{shot} = 1 μJ).

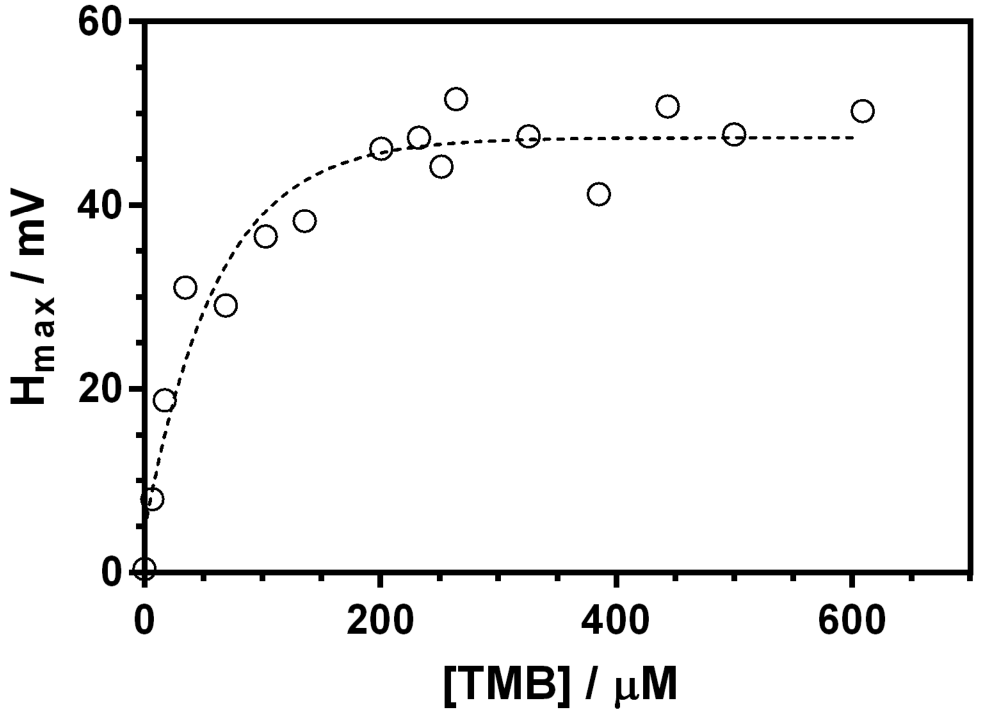

Figure S4. Photoacoustic maximum amplitude for **2** in function of the initial TMB concentration. Two equivalents of TMB reacted with one equivalent of NaClO to maximize the generation of **2**. λ_{exc} = 652 nm.

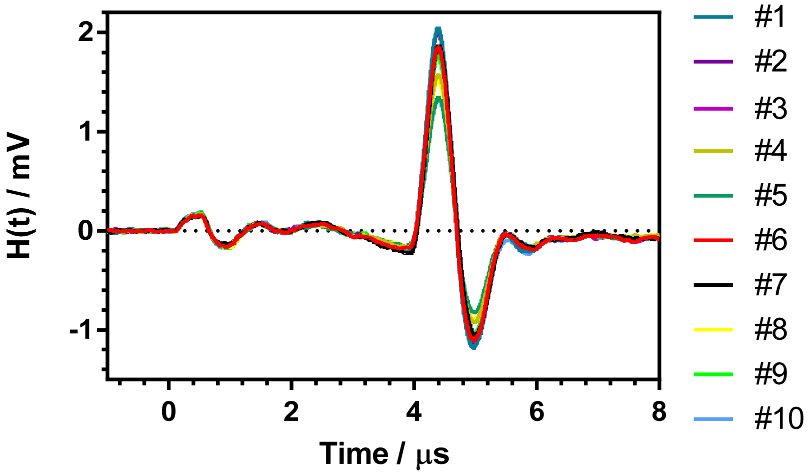

Figure S5. Precision study: Photoacoustic signal for 10 independent replicates of lowest NaClO concentration analytical standard. As visual aid, the replicates with the higher and lower signal are highlighted in blue. [TMB] = 200 μM; [NaClO] = 2 μM; λ_{exc} = 652 nm.

Supporting Information

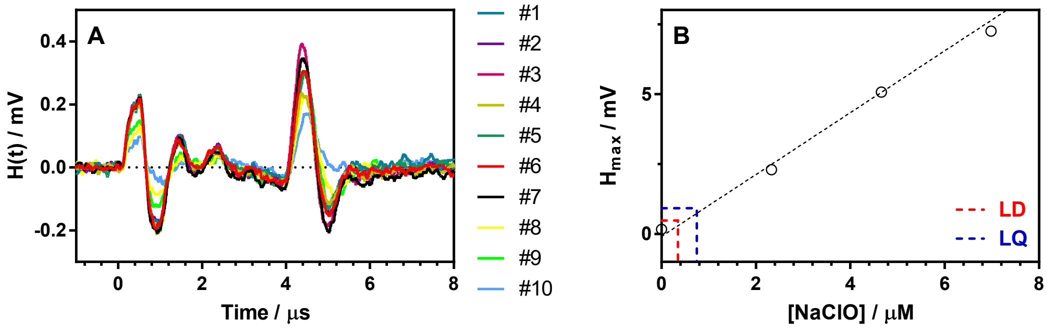

Figure S6. Limit of detection (LOD) and limit of quantification (LOQ) study. (A): Photoacoustic signal for 10 independent replicates of the blank. [TMB] = 200 μM; [NaClO] = 0 μM; λ_{exc} = 652 nm. (B): Interpolation of 3 SD or 10 SD to the calibration curve to obtain the limit of detection (LD) or the limit of quantification (LQ) respectively.

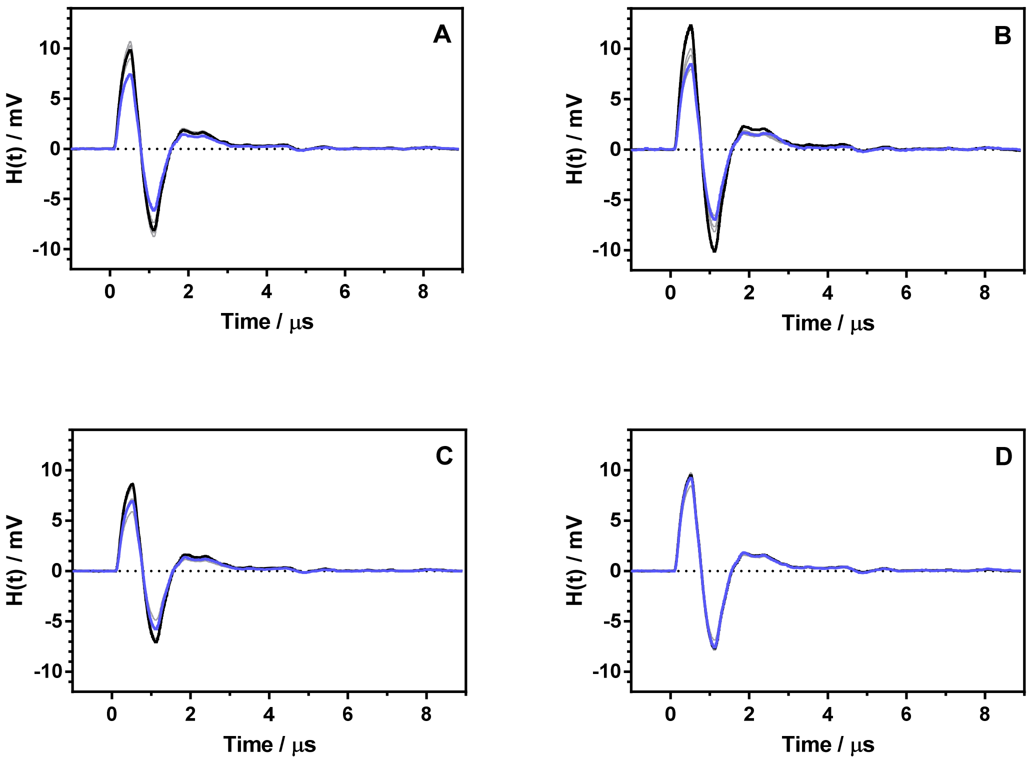

Figure S7. Photoacoustic waveforms for different *E. coli* cell-suspension as a function of the irradiation time (0 to 30 minutes; black to purple lines; lamp power 14.0 mW/cm^2; $\lambda_{irradiation}$ = 459 ± 10 nm). (A): miniSOG-expressing cells. (B): miniSOGQ103L expressing cells. (C): miniSOGQ103V expressing cells. (D): untransformed DH10β cells.

Supporting Information

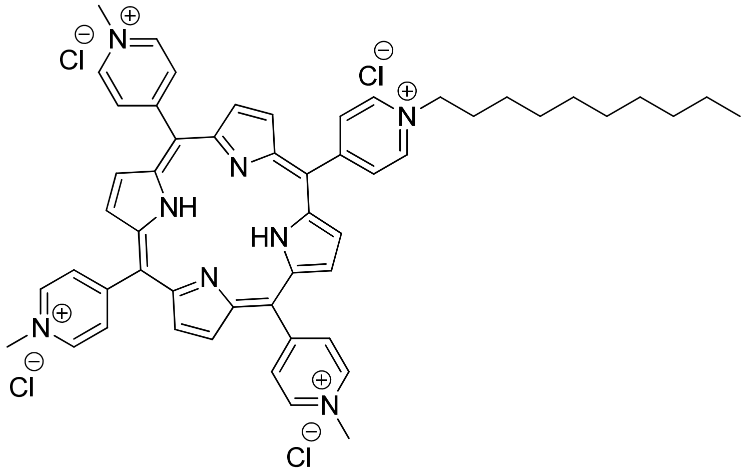

Scheme S3. Chemical structure of 5-mono(N-decyl-4-pyridyl)-10,15,20-tri(N-methyl-4-pyridyl)-21H,23H-porphine tetrachloride (MDPyTMPyP).

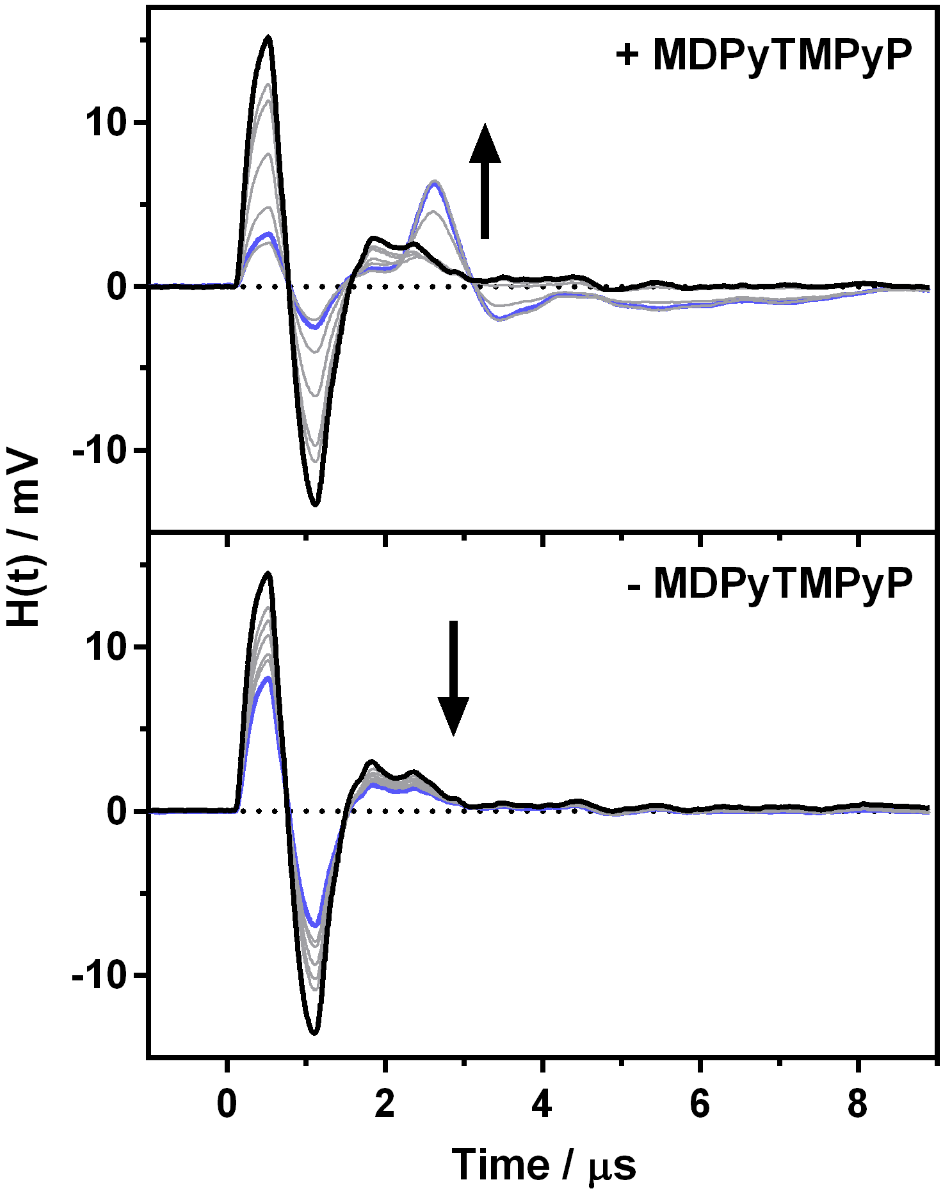

Figure S8. Photoacoustic waveforms for *E. coli* cell-suspension co-incubated with 200 μM TMB and 10 μM MDPyTMPyP (top) or without 10 μM MDPyTMPyP (bottom) as a function of the irradiation time (0 to 45 minutes; black to purple lines; $\lambda_{irradiation}$ = 420 ± 20 nm). The prompt signal at t=0 is due to scattered light hitting the transducer's surface.

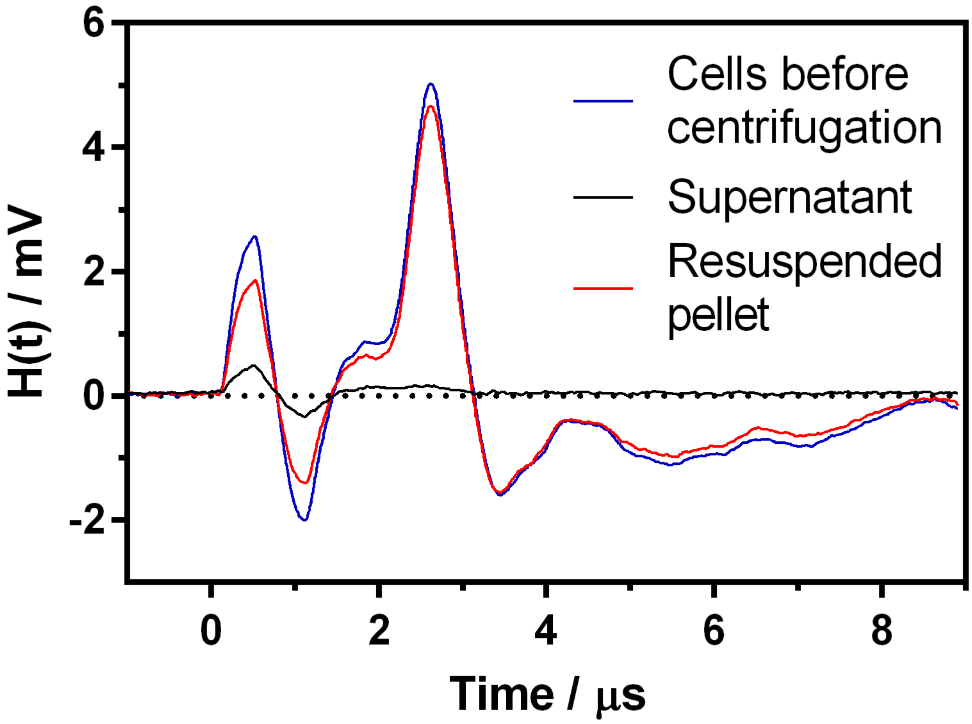

Figure S9. Photoacoustic waveforms for *E. coli* cell-suspension incubated with 200 µM TMB and 10 µM MDPyTMPyP after 45 minutes irradiation (420 ± 20 nm; blue line). Afterwards, the suspension was centrifugated and the photoacoustic waveforms for the supernatant and resuspended pellet were collected too (black and red lines respectively).

Supporting Information

Supporting information References

1. Macrides, T.; Shihata, A.; Kalafatis, N.; Wright, P. A comparison of the hydroxyl radical scavenging properties of the shark bile steroid 5β-scymnol and plant pycnogenols. *IUBMB Life* **1997**, *42*, 1249–1260, doi:10.1080/15216549700203721.

2. Barreto, J. C.; Smith, G. S.; Strobel, N. H. P.; McQuillin, P. A.; Miller, T. A. Terephthalic acid: A dosimeter for the detection of hydroxyl radicals in vitro. *Life Sci.* **1994**, *56*, PL89-PL96, doi:10.1016/0024-3205(94)00925-2.

3. Jankowski, J. J.; Kieber, D. J.; Mopper, K. Nitrate and Nitrite Ultraviolet Actinometers. *Photochem. Photobiol.* **1999**, *70*, 319–328, doi:10.1111/j.1751-1097.1999.tb08143.x.
